# *P14AS* upregulates gene expression in the *CDKN2A/2B* locus through competitive binding to PcG protein CBX7

**DOI:** 10.3389/fcell.2022.993525

**Published:** 2022-09-13

**Authors:** Zhuoqi Li, Juanli Qiao, Wanru Ma, Jing Zhou, Liankun Gu, Dajun Deng, Baozhen Zhang

**Affiliations:** Key Laboratory of Carcinogenesis and Translational Research (Ministry of Education/Beijing), Division of Etiology, Peking University Cancer Hospital and Institute, Beijing, China

**Keywords:** lncRNA, P14AS, CBX7, p16^INK4A^, p14^ARF^, p15^INK4B^

## Abstract

**Background:** It is well known that *P16* ^*INK4A*^, *P14* ^*ARF*^, *P15* ^*INK4B*^ mRNAs, and *ANRIL* lncRNA are transcribed from the *CDKN2A/2B* locus. LncRNA *P14AS* is a lncRNA transcribed from antisense strand of *P14* ^*ARF*^ promoter to intron-1. Our previous study showed that *P14AS* could upregulate the expression level of *ANRIL* and *P16* ^*INK4A*^ and promote the proliferation of cancer cells. Because polycomb group protein CBX7 could repress *P16* ^*INK4A*^ expression and bind *ANRIL*, we wonder whether the *P14AS-*upregulated *ANRIL* and *P16* ^*INK4A*^ expression is mediated with CBX7.

**Results**: In this study, we found that the upregulation of *P16* ^*INK4A*^, *P14* ^*ARF*^, *P15* ^*INK4B*^ and *ANRIL* expression was induced by *P14AS* overexpression only in HEK293T and HCT116 cells with active endogenous *CBX7* expression, but not in MGC803 and HepG2 cells with weak *CBX7* expression. Further studies showed that the stable shRNA-knockdown of *CBX7* expression abolished the *P14AS*-induced upregulation of these *P14AS* target genes in HEK293T and HCT116 cells whereas enforced *CBX7* overexpression enabled *P14AS* to upregulate expression of these target genes in MGC803 and HepG2 cells. Moreover, a significant association between the expression levels of *P14AS* and its target genes were observed only in human colon cancer tissue samples with high level of *CBX7* expression (*n* = 38, *p* < 0.05), but not in samples (*n* = 37) with low level of *CBX7* expression, nor in paired surgical margin tissues. In addition, the results of RNA immunoprecipitation (RIP)- and chromatin immunoprecipitation (ChIP)-PCR analyses revealed that lncRNA *P14AS* could competitively bind to CBX7 protein which prevented the bindings of CBX7 to both lncRNA *ANRIL* and the promoters of *P16* ^*INK4A*^, *P14* ^*ARF*^ and *P15* ^*INK4B*^ genes. The amounts of repressive histone modification H3K9m3 was also significantly decreased at the promoters of these genes by *P14AS* in CBX7 actively expressing cells.

**Conclusions**: CBX7 expression is essential for *P14AS* to upregulate the expression of *P16* ^*INK4A*^, *P14* ^*ARF*^, *P15* ^*INK4B*^ and *ANRIL* genes in the *CDKN2A/2B*locus. *P14AS* may upregulate these genes’ expression through competitively blocking CBX7-binding to *ANRIL* lncRNA and target gene promoters.

## Background

The *CDKN2A/2B* locus on human chromosome 9p21, known as *p15* ^*INK4B*^ *-P14* ^*ARF*^ *-P16* ^*INK4A*^ gene cluster, encodes three tumor suppressor proteins P15, P16, P14. P16 and P15 play as inhibitors of CDK4/6 kinases that control the CDK4/6-RB-E2F pathway. P14 protects P53 and CDKN1A/P21 proteins from degradation through inhibiting the activity of E3-ubiquitylase MDM2 (Serrano et al., 1993; Gil and Peters, 2006; Sherr, 2012). They control G1-S transition of the cell cycle and prevent the development of cancers. Two long noncoding RNA (lncRNA) *ANRIL* and *P14AS* are also transcribed from antisense strand of this gene cluster. While these tumor suppressors are frequently inactivated or downregulated by genetic and epigenetic mechanisms (Zhao et al., 2016; The ICGC/TCGA Pan-Cancer Analysis of Whole Genomes Consortium, 2020; Popov and Gil, 2010), oncogenic *ANRIL* and *P14AS* are often overexpressed in many human cancers (Lou et al., 2020; Ma et al., 2020). Our and other’s works demonstrated that *ANRIL* and *P14AS* could regulate these tumor suppressor expression (Yu et al., 2008; Aguilo et al., 2011; Kotake et al., 2011; Ma et al., 2020). However, the regulatory mechanisms for these lncRNAs are far from clear.

Polycomb group (PcG) proteins are well known transcription repressors for the *CDKN2A/2B* locus through inducing repressive histone modifications, including H3K27m3 and H3K9m3, subsequently causing condensation of the local chromatin structures (Li et al., 2010; Yap et al., 2010; Laugesen et al., 2019). A recent study demonstrated that lncRNA was required for proper localization of core proteins (such as EZH2 and SUZ12) in polycomb repressive complex 2 (PRC2) to chromatin targets in human pluripotent stem cells (Long et al., 2020). *ANRIL* was reported to downregulate *P15^INK4B^* and *P16^INK4A^* expression by interacting with PRC1 and PRC2 proteins (Yu et al., 2008; Kotake et al., 2011). *ANRIL* could bind to SUZ12 and CBX7 and recruit them to the *P15* ^*INK4B*^ *-P14* ^*ARF*^ *-P16* ^*INK4A*^ locus to promote the formation of H3K27m3 and repress these genes’ transcription (Yap et al., 2010; Laugesen et al., 2019). In our previous work, we found that CBX7, a PRC1 member, was able to inhibit the expression of *P16^INK4A^* by inducing H3K9m3 (Li et al., 2010). Recently, we have reported that the novel lncRNA *P14AS* could upregulate the expression of the *P16* ^*INK4A*^ gene and lncRNA *ANRIL* through AUF1-binding and promote the proliferation of colon cancer cells in a *P16* ^*INK4A*^ expression-independent pattern (Ma et al., 2020). Whether the *P14AS* regulates these gene expression through CBX7-mediated pathway is unknown.

In the present work, we studied the impact of CBX7 on effect of *P14AS* on transcription of genes in the *CDKN2A/2B* locus systemically in details.

## Materials and methods

### Cell culture and tissue samples

The human cell line HEK293T was kindly provided by Professor Yasuhito Yuasa at Tokyo Medical and Dental University. HCT116 cell line was kindly provided by Professor Yuanjia Chen at Peking Union Medical College Hospital. MGC803 was kindly provided by Professor Yang Ke and HepG2 cell line was kindly provided by Professor Qingyun Zhang at Peking University Cancer Hospital and Institute. The colon cancer (CC) tissues and paired normal tissues from the surgical margin (SM, >5 cm from cancer lesion) from CC patients were collected and stored at −70°C at Peking University Cancer Hospital from 2004 to 2011 (Zheng et al., 2015; Ma et al., 2020). Research protocols were approved by the Institutional Review Board of the Peking University Cancer Hospital and Institute, China. Clinical and histopathological data for each patient were obtained according to approved institutional guidelines.

### Plasmid construction and transfection

The 1043-nt *P14AS* lentiviral vector was constructed in PCDH-CMV-EF1a-copGFP-T2A-Puro lentiviral vector by Syngen tech Co., Ltd. (Beijing, China) (Ma et al., 2020). The full length of *CBX7* cDNA was inserted into expression vector pEGFP-C1. The shRNA sequence (caaag tacag cacgt ggga) targeting *CBX7* (accession number XM_066324) was constructed using pGFPU6/Neo vector (GenePharma Company, Shanghai). The scramble shRNA (gttct ccgaa cgtgt cacgt) was used as negative control (Li et al., 2010).

### RNA extract and realtime PCR

The total RNA of cell lines and tissues were isolated using Direct-zol RNA MiniPrep Kit (ZYMO Research, USA) and then reverse transcripted into cDNA using TransScript First-Strand cDNA Synthesis SuperMix (TransGen Biotech, China) according to the manufacturer’s instructions. Next, quantitative RT-PCR (qRT-PCR) was performed using the FastStart Universal SYBR Green Master (ROX) (Roche, Germany) on an ABI-7500 Fast system (Applied Biosystems). *GAPDH* and *ALU* were used as the endogenous reference genes for the cultured cell lines and tissues, respectively (Zheng et al., 2015). Each sample was quantitatively analyzed in triplicates. The relative expression levels of these genes were determined using the typical ΔΔCt method. Total tissue samples were divided into two groups with low- and high-level of *CBX7* expression using the median as the cutoff value. The differences in target gene expression between these two groups were analyzed with Student’s t-test and plotted in GraphPad Prism 9.0. *p* < 0.05 was considered statistically significant (N.S: not significant. *: *p* < 0.05. **: *p* < 0.01). Primer sequences were showed in Supplementary Table S1.

### Western blot

Total protein was extracted from cultured cells using RIPA buffer. The primary polyclonal antibody against P14 (ab3648, Abcam), P15 (ab53034, Abcam), P16 (ab81278, Abcam), CBX7 (ab21873, Abcam), GAPDH (60004-1, ProteinTech), or green fluorescent protein (GFP) (ProteinTech, 50430-2-AP; China) was applied at 1:5,000 to 1:1,000 dilutions. The signals were visualized using the Enhanced Chemiluminescence Kit (Millipore) and Alpha Imager system.

### RNA immunoprecipitation assay

The RIP assay was carried out using Magna RIP RNA-Binding Protein Immunoprecipitation Kit (Sigma) according to the manufacturer’s instructions. Total CBX7-binding RNAs were immunoprecipitated and extracted using anti-CBX7 antibody (ab21873, Abcam). cDNA was synthesized from the RIP-RNAs using random primers, and gene-specific quantitative PCR was then performed using FastStart Universal SYBR Green Master (ROX) (Roche, Germany). *18S rRNA* was used as positive and reference control to calculate the enrichment of lncRNA. The qPCR products were analyzed by agarose gel or PAGE gel to detect product size and specificity of amplification.

### Chromatin immunoprecipitation assay

ChIP assays were performed and analyzed essentially as described (Li et al., 2010). Rabbit anti-CBX7, rabbit anti-H3K9m3 (07-442; Sigma-Aldrich), and rabbit anit-H3K27m3 (61017; Active Motif) antibodies were used to precipitate endogenous CBX7 and modified histone proteins and the binding chromatin. The enrichment of specific genomic regions was assessed relative to Input DNA. IgG antibody was used as negative control (PP64B, Sigma-Aldrich). Each ChIP experiment was quantitatively analyzed in triplicates. The qPCR products were analyzed by agarose gel or PAGE gel to detect product size and specificity of amplification. Primer sets and annealing temperature used were listed in Supplementary Table S1.

## Results

### *P14AS* upregulates the expression of *P15* ^*INK4B*^ *-P14* ^*ARF*^ *-P16* ^*INK4A*^ locus depending on CBX7

**FIGURE 1:**
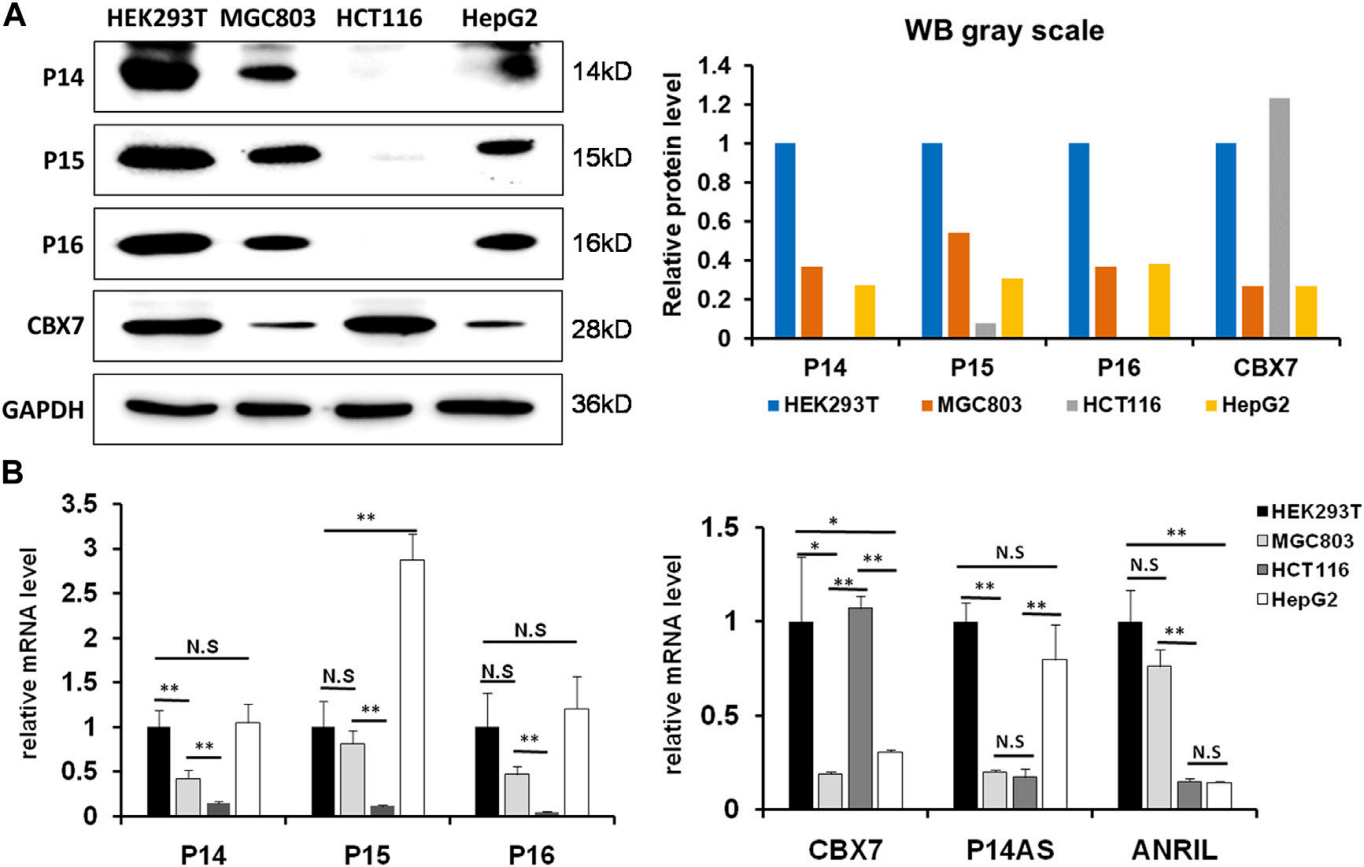
The status of basal CBX7, *P16* ^*INK4A*^ *, P14* ^*ARF*^, *P15* ^*INK4B*^ *, P14AS* and *ANRIL* expression in human cell lines. **(A)** the results of Western blot; **(B)** qRT-PCR analyses. The relative mRNA levels are presented as mean ± SD. N.S: not significant. *: *p* < 0.05. **: *p* < 0.01 in Student’s t-test.

We previously reported that *P14AS* upregulated *P16* ^*INK4A*^ and *ANRIL* expression, and that CBX7 downregulated *P16* ^*INK4A*^ expression through inducing H3K9me3 modification (Li et al., 2010; Ma et al., 2020). To study the effect of CBX7 on *P14AS-*induced *P16* ^*INK4A*^ and *ANRIL* expression, we selected proper cell lines through analyzing the basal expression states of CBX7 and its target genes at first. In Western blot analysis, the amount of endogenous CBX7 protein was much greater in HEK293T and HCT116 cells than in MGC803 and HepG2 cells (Figure 1A). The results of qRT-PCR confirmed the Western blot experiment (Figure 1B). No P16 and P14 proteins were detected in HCT116 cells.

**FIGURE 2:**
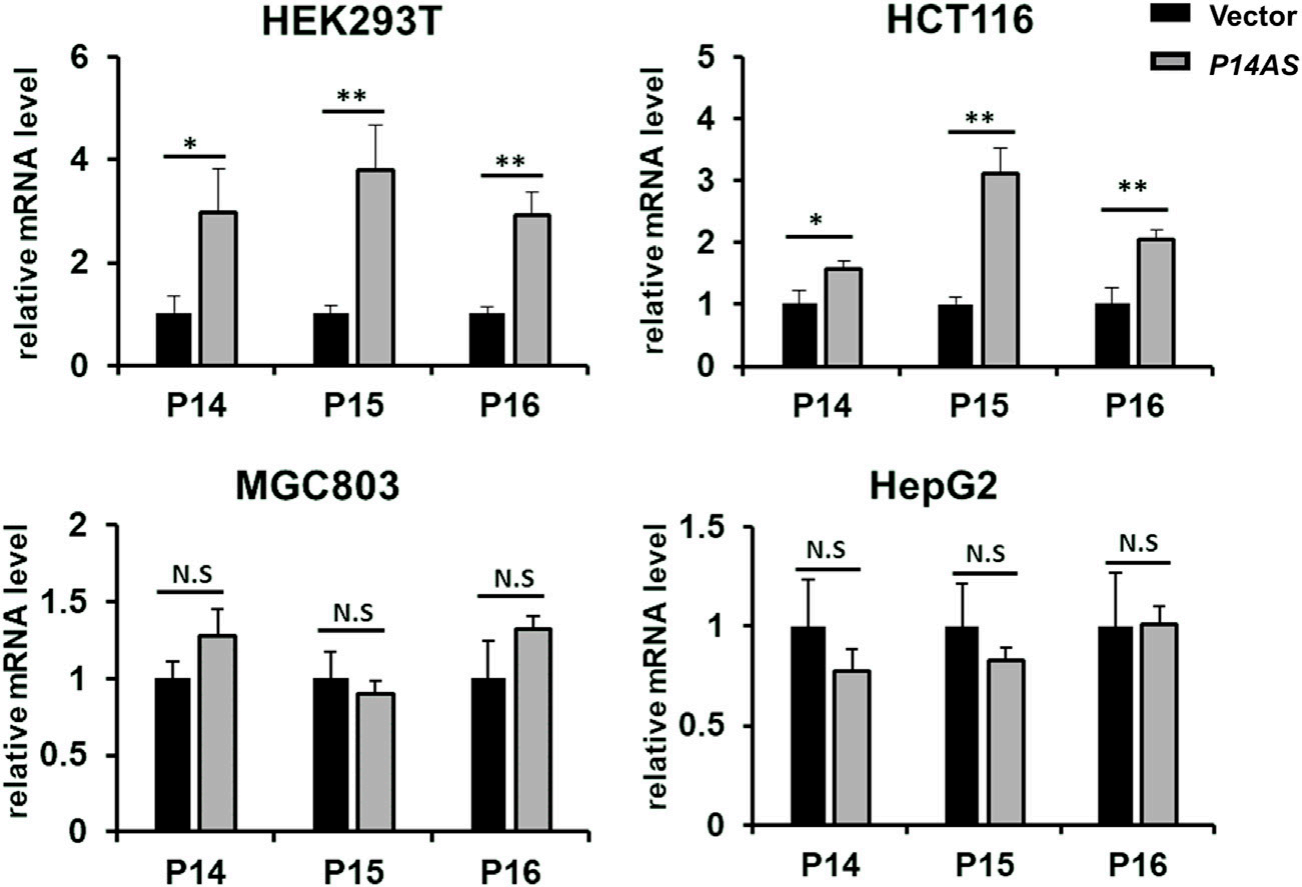
Effect of transient *P14AS* overexpression on the levels of *P14* ^*ARF*^ *, P16* ^*INK4A*^ and *P15* ^*INK4B*^ expression in qTR-PCR analyses.

Then, we compared the effect of *P14AS* on the upregulation of *P16* ^*INK4A*^ *, P14* ^*ARF*^ and *P15* ^*INK4B*^ expression in these cell lines with high and low *CBX7* expression. The results showed that transient *P14AS* overexpression obviously upregulated the expression levels of *P16* ^*INK4A*^ *, P14* ^*ARF*^ and *P15* ^*INK4B*^ only in HEK293T and HCT116 cells with active *CBX7* expression, but not in MGC803 and HepG2 cell lines with low *CBX7* expression (Figure 2). These results imply that upregulation of *P16* ^*INK4A*^ *, P14* ^*ARF*^ and *P15* ^*INK4B*^ by *P14AS* might be dependent on active *CBX7* expression.

**FIGURE 3:**
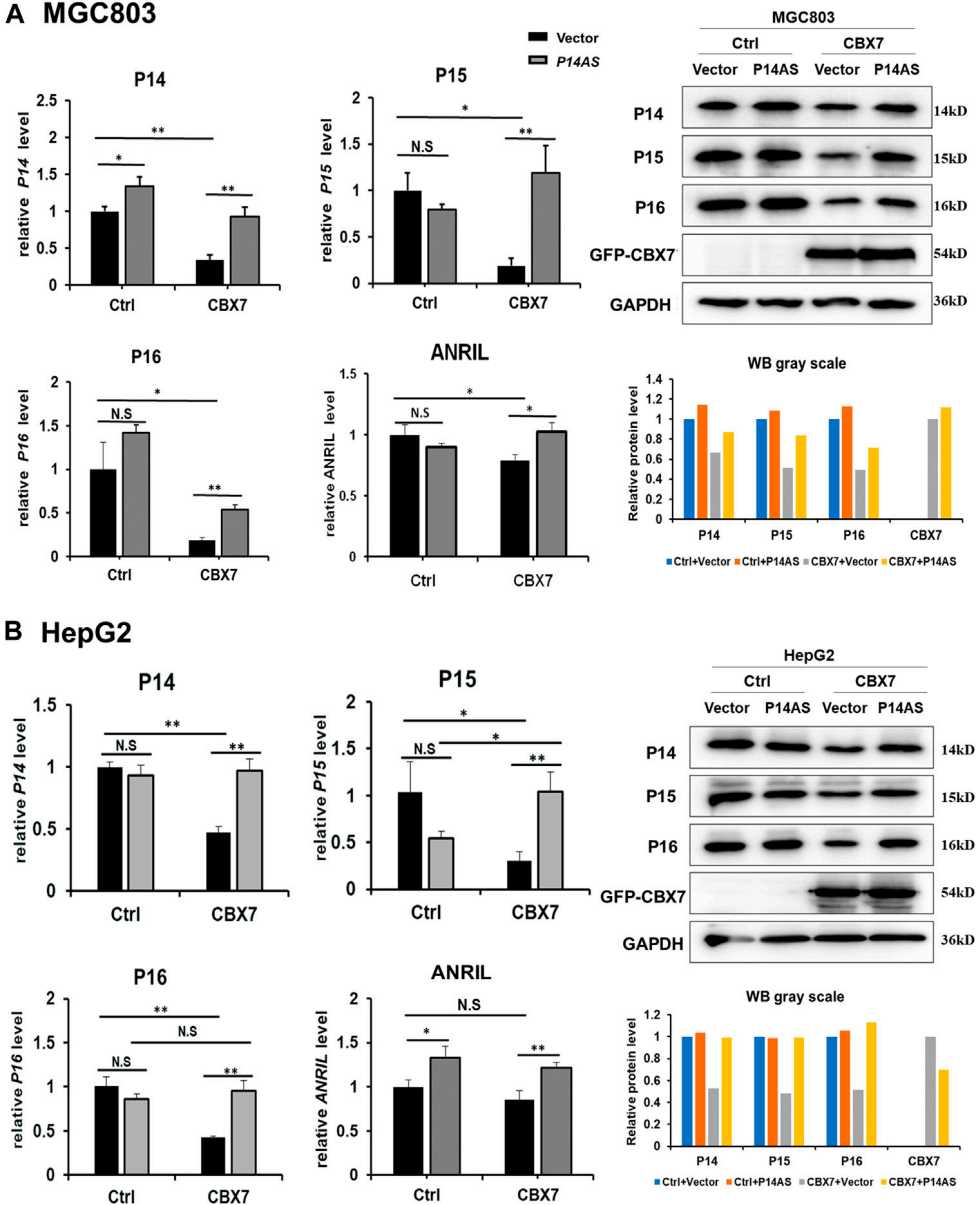
Increasing *CBX7* expression enables *P14AS* to upregulate the *P14* ^*ARF*^, *P15* ^*INK4B*^ *, P16* ^*INK4A*^ expression levels in MGC803 and HepG2 cells with weak basal *CBX7* expression in qRT-PCR (left charts for the relative RNA level) and Western blot analyses (right image and chart for the amounts of proteins). **(A)** MGC803; **(B)** HepG2 cells.

To clarify the role of CBX7 in the upregulation effect of *P14AS* on *P16* ^*INK4A*^ *, P14* ^*ARF*^ *, P15* ^*INK4B*^ and *ANRIL*, the *CBX7* expression level was artificially increased in MGC803 and HepG2 cells by transfection of the *CBX7* expression vector and decreased in HEK293T and HCT116 cells by transfection of the shR-CBX7 vector, and then the cells were co-transfected with *P14AS* expression or empty vectors (Supplementary Figure S1). As expected, both the qRT-PCR and Western blot results showed that *CBX7* overexpression enabled *P14AS* to obviously upregulate *P16* ^*INK4A*^ *, P14* ^*ARF*^ *, P15* ^*INK4B*^ and *ANRIL* expression in the co-transfected MGC803 and HepG2 cells (Figures 3A,B). Overexpressed *CBX7* could inhibit the expression of these genes at different level.

**FIGURE 4:**
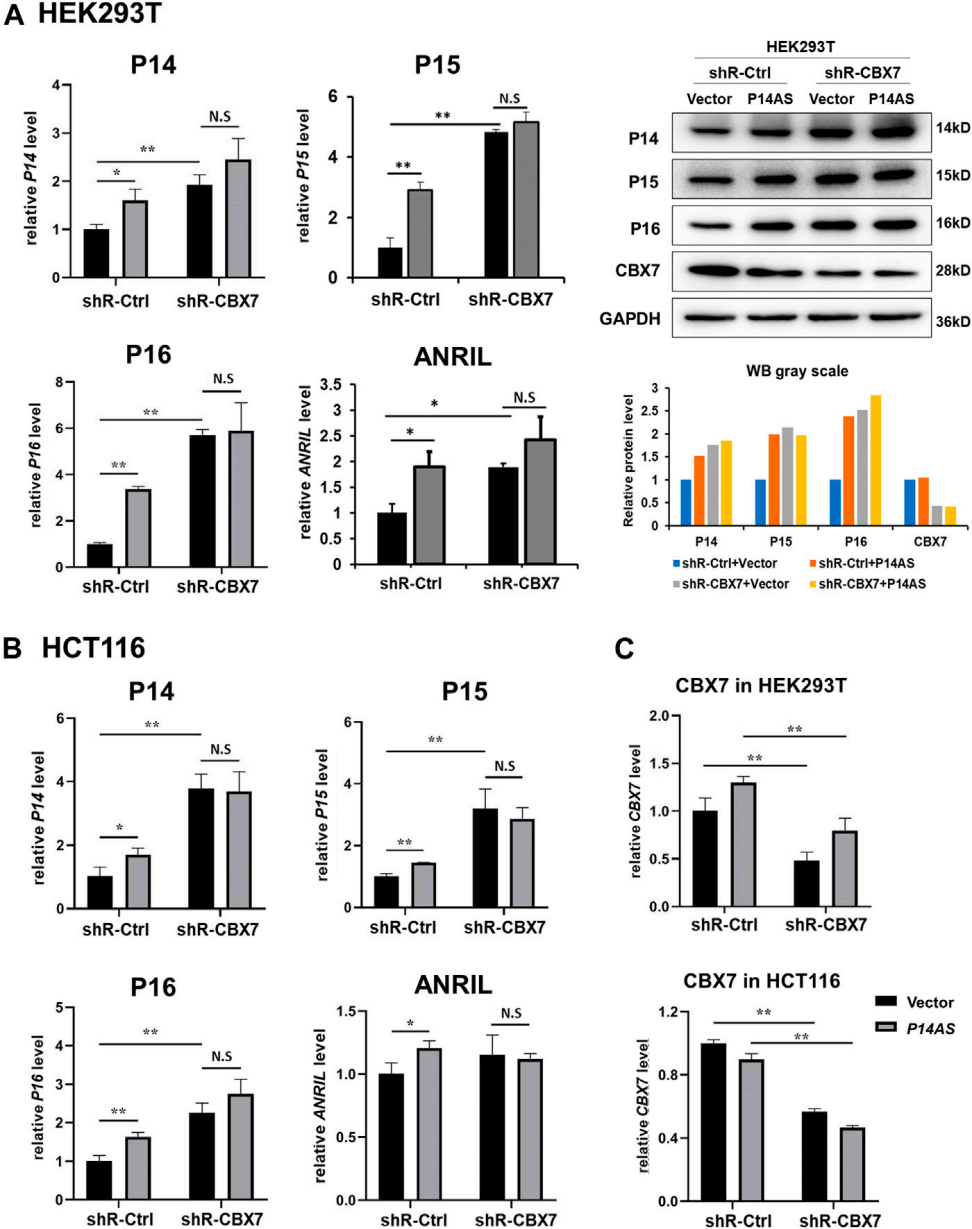
Downregulation of *CBX7* expression disables *P14AS* to upregulate the *P14* ^*ARF*^, *P15* ^*INK4B*^ *, P16* ^*INK4A*^ expression levels in HEK293T and HCT116 cells with active basal *CBX7* expression in qRT-PCR and Western blot analyses. **(A)** HEK293T; **(B)** HCT116 cells; **(C)** the efficiency of CBX7 knockdown in HEK293T and HCT116.

In contrast, the upregulating effects of *P14AS* overexpression were almost disappeared when endogenous *CBX7* expression was stably knocked down by shR-CBX7 treatment in HEK293T and HCT116 cells co-transfected with *P14AS* (Figures 4A,B).

All the above results support the hypothesis that *P14AS* upregulates the expression of these target genes in an active *CBX7* expression-dependent pattern.

### Coexpression of *P14AS* with *P14* ^*ARF*^ and *P16* ^*INK4A*^ genes in human colon cancer tissues with high *CBX7* expression level

**FIGURE 5:**
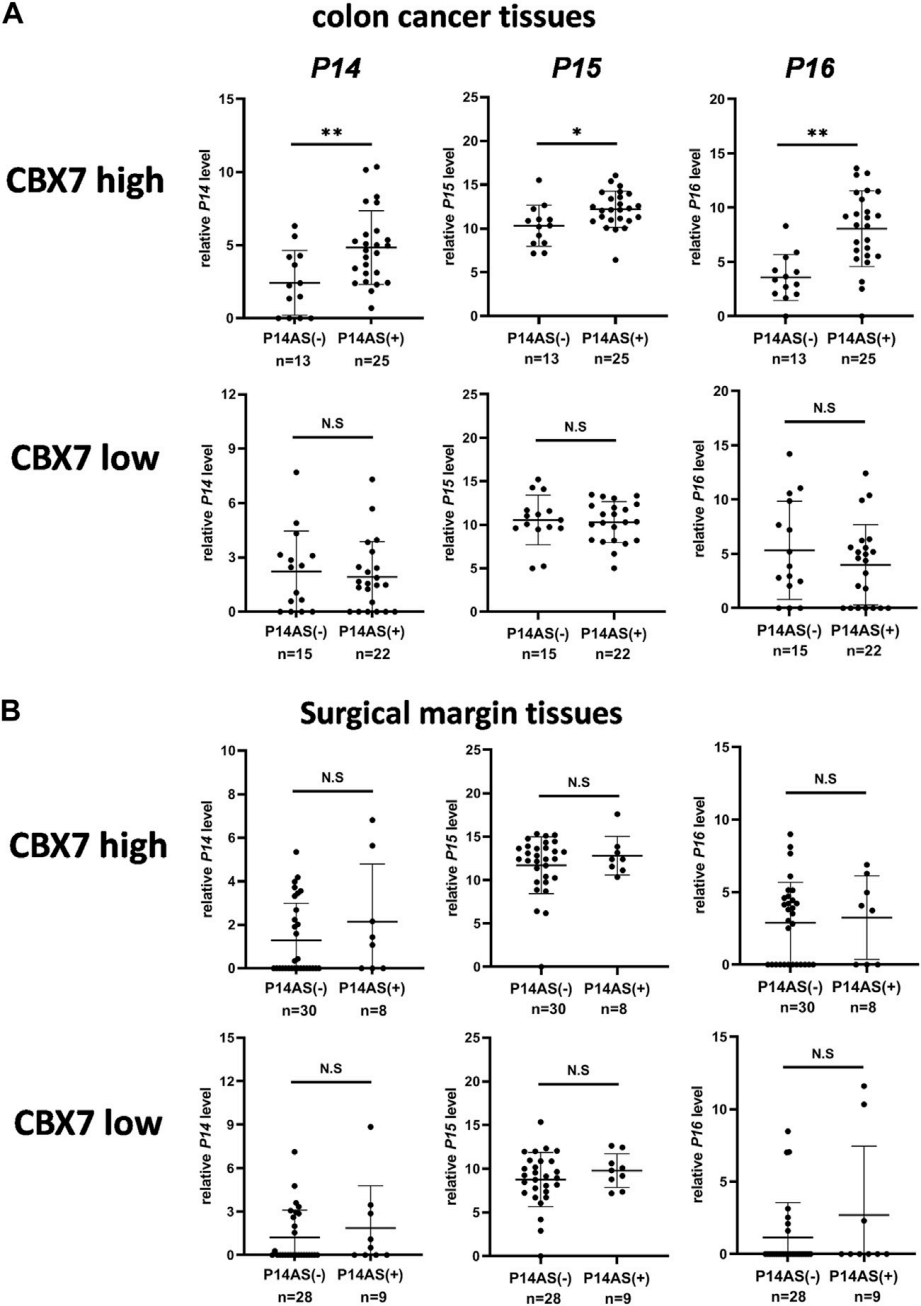
Coexpression of *P14AS* with *P14* ^*ARF*^, *P15* ^*INK4B*^ and *P16* ^*INK4a*^ genes in human colon cancer tissues with high *CBX7* expression. **(A)** Colon cancer tissues; **(B)** Surgical margin tissues. N.S: not significant. *: *p* < 0.05. **: *p* < 0.01.

To study whether the above *CBX7* expression-dependent effect of *P14AS* on the upregulation of *P14* ^*ARF*^, *P15* ^*INK4B*^ *, P16* ^*INK4A*^ expression occurs in human tissues, we determined the levels of *P14* ^*ARF*^, *P15* ^*INK4B*^ *, P16* ^*INK4A*^, *P14AS* and *CBX7* in colon cancer and paired normal surgical margin tissues from patients (n = 75) by qRT-PCR. Then, these samples were divided into the *CBX7* expression-high and -low groups, according to the median *CBX7* expression level. As expected, the expression levels of *P16* ^*INK4A*^ and *P14* ^*ARF*^ were significantly higher in the *P14AS* expression-positive (+) samples than in the *P14AS* expression-negative (−) samples only within the *CBX7* high colon cancer group, but not within the *CBX7* low colon cancer group (Figure 5A). In addition, no significant difference in these genes’ expression levels was observed between the *P14AS* expression-positive and expression-negative samples in the paired normal samples (Figure 5B), suggesting a tumor-specific effect of CBX7 on *P14AS* functions. These results confirm the hypothesis that *CBX7* expression is essential for *P14AS* to regulate *P16* ^*INK4A*^, *P14* ^*ARF*^ and *P15* ^*INK4B*^ expression in cancer tissues.

### *P14AS* competitively binds to CBX7 and prevents the binding of CBX7 to *ANRIL* lncRNA and *P16* ^*INK4A*^ *, P14* ^*ARF*^ *and P15* ^*INK4B*^ promoter DNA

**FIGURE 6:**
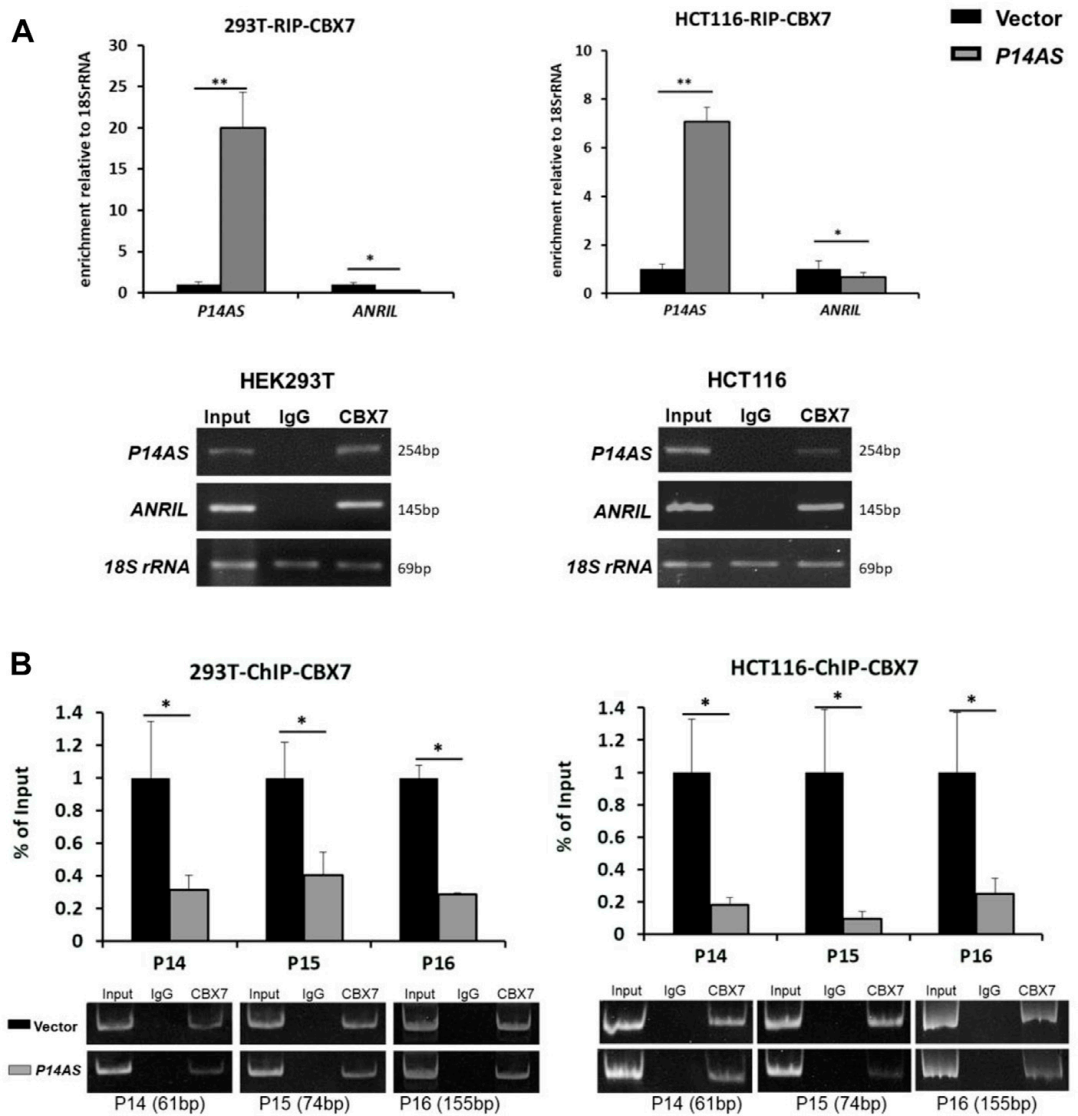
*P14AS* competitively binds to CBX7 and prevents the binding of CBX7 to ANRIL and to *P14* ^*ARF*^, *P15* ^*INK4B*^, *P16* ^*INK4A*^ promoters. **(A)** Levels of *P14AS* and *ANRIL* binding to endogenous CBX7, as determined by RIP-qPCR analyses; and **(B)** binding of gene promoters to endogenous CBX7, as determined by ChIP-qPCR analyses. The experiments were repeated using both HEK293T and HCT116 cell lines.

To explore the molecular mechanisms of CBX7 affecting the functions of *P14AS* in the regulation of these target genes, we conducted qPCR analysis based on RIP and ChIP assays using anti-CBX7 antibody. The RIP-qPCR results showed that native CBX7 directly bound not only to lncRNA *P14AS*, but also to lncRNA *ANRIL*. In the HEK293T and HCT116 cells with *P14AS* overexpression, the CBX7-*P14AS* protein-RNA binding was increased, while the CBX7-*ANRIL* protein-RNA binding was decreased relative to the vector control cells (Figure 6A). Furthermore, the ChIP-qPCR results showed that native CBX7 directly bound to promoter DNA of these target genes. Notably, the protein-DNA bindings of CBX7-promoters were also decreased in the *P14AS* overexpression cells (Figure 6B). Together, these results indicated that *P14AS* could competitively bind to CBX7, which consequently prevented the CBX7-*ANRIL* and CBX7-promoter bindings and upregulated the expression levels of these target genes.

**FIGURE 7:**
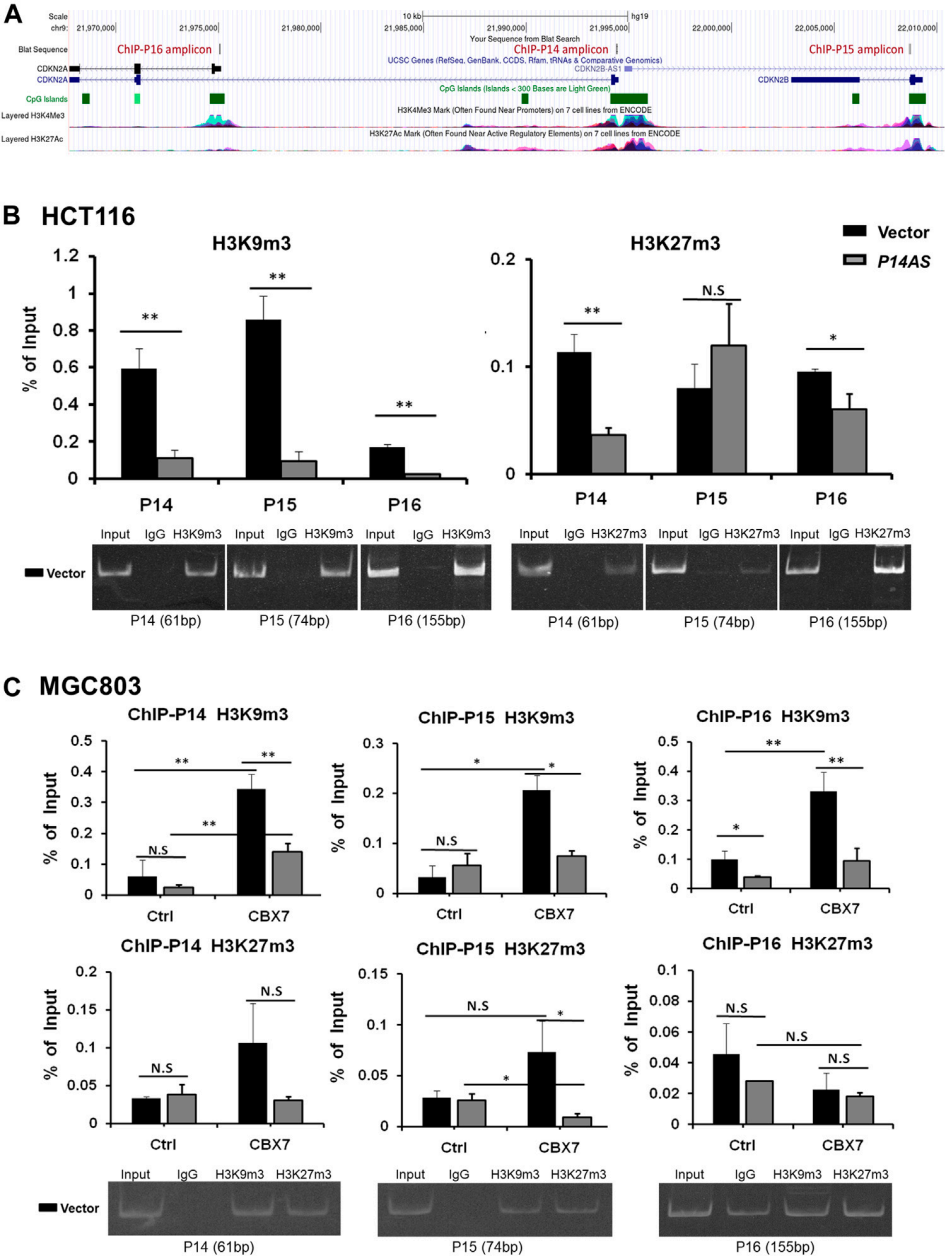
Inhibition of repressive histone modifications at promoters of *P14* *^ARF^*, *P15* *^INK4B^*, and *P16* ^*INK4A*^ by *P14AS* is CBX7-dependent, as determined by ChIP-qPCR analyses. **(A)** The ChIP-qPCR fragments and histone modifications within the locus, a screenshot from UCSC Genome Browser; **(B)** The levels of H3K9m3 and H3K27m3 at the three target genes promoters in HCT116 cells stably transfected with the *P14AS* or empty vector; **(C)** The levels of H3K9m3 and H3K27m3 at the three genes’ promoters in MGC803 cells cotransfected with *P14AS* and CBX7 or empty control vectors. The gel pictures show the size and specificity of qPCR products.

In addition, we checked the status of histone modifications on these gene promoters in ChIP-qPCR analysis. We found that *P14AS* overexpression significantly decreased the recruitment of trimethylation of both H3K9 and H3K27 (H3K9m3 and H3K27m3) at these genes’ promoters in HCT116 cells with active *CBX7* expression (Figure 7A), but not in MGC803 cells with low *CBX7* expression (Figure 7B in the first two columns in Ctrl cells). However, in the MGC803 cells with enforced *CBX7* overexpression, *P14AS* overexpression decreased the recruitment of H3K9m3 at these genes’ promoters (Figure 7B, in the last two columns in *CBX7* overexpressed cells), The enforced *CBX7* overexpression could also increase the recruitment of H3K9m3 at these genes’ promoters (Figure 7B, in the two black bars in the same panel), which was consistent with the repressive effects of CBX7 on these genes. However, the recruitments of H3K27m3 at these promoters were not changed as obviously as H3K9m3.

These results illustrated that inhibition of repressive histone modifications by *P14AS* is also CBX7 expression-dependent.

## Discussion

It has been reported that both lcnRNA *ANRIL* and promoters of *P14* ^*ARF*^, *P15* ^*INK4B*^, *P16* ^*INK4A*^ can directly bind to the PcG proteins including CBX7, and form heterochromatin surrounding the *CDKN2A/2B* locus, which leads to the repression of *P15* ^*INK4B*^ expression (Yu et al., 2008; Aguilo et al., 2011; Kotake et al., 2011). PcG proteins such as CBX7 have also been reported to repress *P16* ^*INK4A*^ transcription (Gil et al., 2004; Li et al., 2010; Yap et al., 2010). In our recent study, we reported that lncRNA *P14AS* transcribed from the antisense strand of *P14* ^*ARF*^ promoter to intron-1 could directly upregulate the expression of *ANRIL*-*CDKN2A/2B* locus (Ma et al., 2020). Here, we further show that *P14AS* may act as *cis*-activator to increase these genes’ expression in a CBX7-binding-dependent pattern.

*ANRIL* contains 19–21 reported exons over a 126 kb region franking the *P15* ^*INK4B*^ gene (Yap et al., 2010). *P14AS* is transcribed from the upstream of *ANRIL* and there is a 79-nt complete overlap between its exon-3 and *ANRIL* exon-1 (Ma et al., 2020). We wonder that *P14AS* might be an isoform of *ANRIL* but use different transcription start site. However, they have distinct effects on the expression regulation of their host genes and are likely to contribute to the concerto of transcription regulation. Recently, the splicing variants of *ANRIL* were reported to exert opposing effects on endothelial cell activities associated with coronary artery disease (Cho et al., 2020). And truncated isoforms of *ANRIL* were reported to be overexpressed in bladder cancer and differently correlated with the expression of the *P14* *^ARF^*, *P15* *^INK4B^*, and *P16* ^*INK4A*^ genes (Hoffmann et al., 2015). Circular *ANRIL* isoforms could switch from repressors of *CDKN2B* to activators of *CDKN2A* and change EZH2 localization during *RAF1* oncogene-induced senescence (Muniz et al., 2021). Although we found that total *ANRIL* expression is regulated by the CBX7-*P14AS* binding, which *ANRIL* isoforms’ expression may be affected by the CBX7-*P14AS* binding is worthy of further study.

AUF1 is a member of RNA binding proteins family and play roles in directing RNA decay and translation at post-transcription stage (White et al., 2017). AUF1 was also identified as an important destabilizer for *P16* ^*INK4A*^ mRNA and thereby influencing cell senescence (Chang et al., 2010). In our previous study, we reported *P14AS* could competitively bind with AUF1 and decreased the AUF1-*ANRIL/P16* ^*INK4A*^ RNA interaction (Ma et al., 2020). Here, we further verified *P14AS* could also competitively bind to CBX7. The overexpression of *P14AS* could also inhibit the repressive histone modifications, including H3K9m3 and H3K27m3, within the *CDKN2A/2B* locus, through inhibiting the CBX7 recruitment, and consequently lead to the upregulation of gene transcription. These results imply that lncRNA may interact with different transcription factors or RNA binding proteins to regulate the gene expression at both transcription and post-transcription level.

We are still at a very early stage of understanding the functions of most lncRNAs. There are a number of existing models to illustrate the principles of lncRNA regulation. Interactions between loops of lncRNAs and transcription factors have been suggested to be important in maintaining promoters in an accessible status to allow for transcription complex interactions (Kung and Lee, 2013; Laugesen et al., 2019; Long et al., 2020). Enhancers generally contain a low-proportion of CpG sites and active enhancers are often associated with H2A.Z replacement and formation of H3K4m1 and H3K27ac (Calo and Wysocka, 2013; Core et al., 2014). According to the publicly available ENCODE database, the *P14AS-*transcribed region matches the typical enhancer location, lying between the mammalian-wide interspersed repeat (*MIR*) element and the CpG island in the *P14^ARF^* promoter (Supplementary Figure S2, highlighted in light-blue). Our recent work also showed that there was a loop between the *P14AS-*transcribed region and *P16^INK4A^* promoter region (unpublished data). This suggests that *P14AS* may be an enhancer lncRNA.

In addition, formation of repressive H3K27m3 and active H3K4m3 have been recognized in embryonic stem cells to be a poised pattern of histone modifications in chromatin of bivalent genes (such as *P16^INK4A^*) (Liu et al., 2016; Kumar et al., 2021). H3K27m3, H3K9m3 and H3K4m3 are the main tri-methylation modifications involved in the formation of “bivalent domains”, which is a unique chromatin region consisting of two histone tri-methylation modifications and plays a vital regulatory role in the differentiation of various stem cell systems (Sun et al., 2022). Among the enriched genes in differentiation process of hepatic stem/progenitor cells, the *CDKN2A* gene was upregulated and exhibiting bivalent domains within 2 kb of transcription start site (Kanayama et al., 2019). The *P14AS*-transcribed region is also a poised PcG responsive element (PRE) region. This implies that the *P14AS* may play a role in maintaining the accessibility to the flanking promoter region for transcription factor complexes.

It is generally considered that the *P16* ^*INK4A*^ *, P14* ^*ARF*^, *P15* ^*INK4B*^ genes at 9p21.3 locus should not be simultaneously silenced in normally differentiated cells. More evidences suggest that these genes provide an important check and balance system that is essential for controlling the G1→S phase transition in cell cycle and that the dysfunctions of these genes play crucial roles in the malignant transformation of human cells (Krimpenfort et al., 2007; Leong et al., 2009). P16 is a weakly expressed nucleic protein in normal cells and long-term high *P16* ^*INK4A*^ expression will promotes cells to enter senescence. Importantly, inactivity of *P16* ^*INK4A*^ is one of the most frequent events in many kinds of human tumors (LaPak and Burd, 2014). Therefore, the regulation of *P15* ^*INK4B*^ *-P14* ^*ARF*^ *-P16* ^*INK4A*^ genome locus maybe a precise and complex process and essential to maintain the coordinated balance between tumor suppression and aging. There are numerous advances in understanding these genes’ functions and regulation since their importance was detected almost 40 years ago. However, the underlying mechanisms of their regulation as well as the coordinated expression patterns of these genes are not yet fully understood. Here we provided a new clue that lncRNA *P14AS* could competitively bind to CBX7 and reduce the repressive histone modifications of this gene cluster region. It might play roles in modifying local genome structure and the precise regulation of the expression of these genes.

## Conclusion

LncRNA *P14AS* can competitively bind to CBX7 and reduce the binding of CBX7 to lncRNA *ANRIL* and the promoters of *P16* ^*INK4A*^, *P14* ^*ARF*^ and *P15* ^*INK4B*^ genes, which results in their up-regulation.

## Data Availability

The original contributions presented in the study are included in the article/Supplementary Material; further inquiries can be directed to the corresponding authors.
