# Corrigendum: P14AS upregulates gene expression in the CDKN2A/2B locus through competitive binding to PcG protein CBX7

**DOI:** 10.3389/fcell.2022.1062931

**Published:** 2022-12-09

**Authors:** Zhuoqi Li, Juanli Qiao, Wanru Ma, Jing Zhou, Liankun Gu, Dajun Deng, Baozhen Zhang

**Affiliations:** Division of Etiology, Peking University Cancer Hospital, Beijing, China

**Keywords:** LncRNA, P14AS, CBX7, P16INK4A, P14ARF, P15INK4B

In the published article, there was an error in Figure 6 as published. Part of the gel picture was not displayed. The corrected Figure 6 and its full caption “*P14AS* competitively binds to CBX7 and prevents the binding of CBX7 to *ANRIL* and *P14* ^*ARF*^, *P15* ^*INK4B*^, *P16* ^*INK4A*^ promoters” are shown at the end of the article.

**FIGURE 6:**
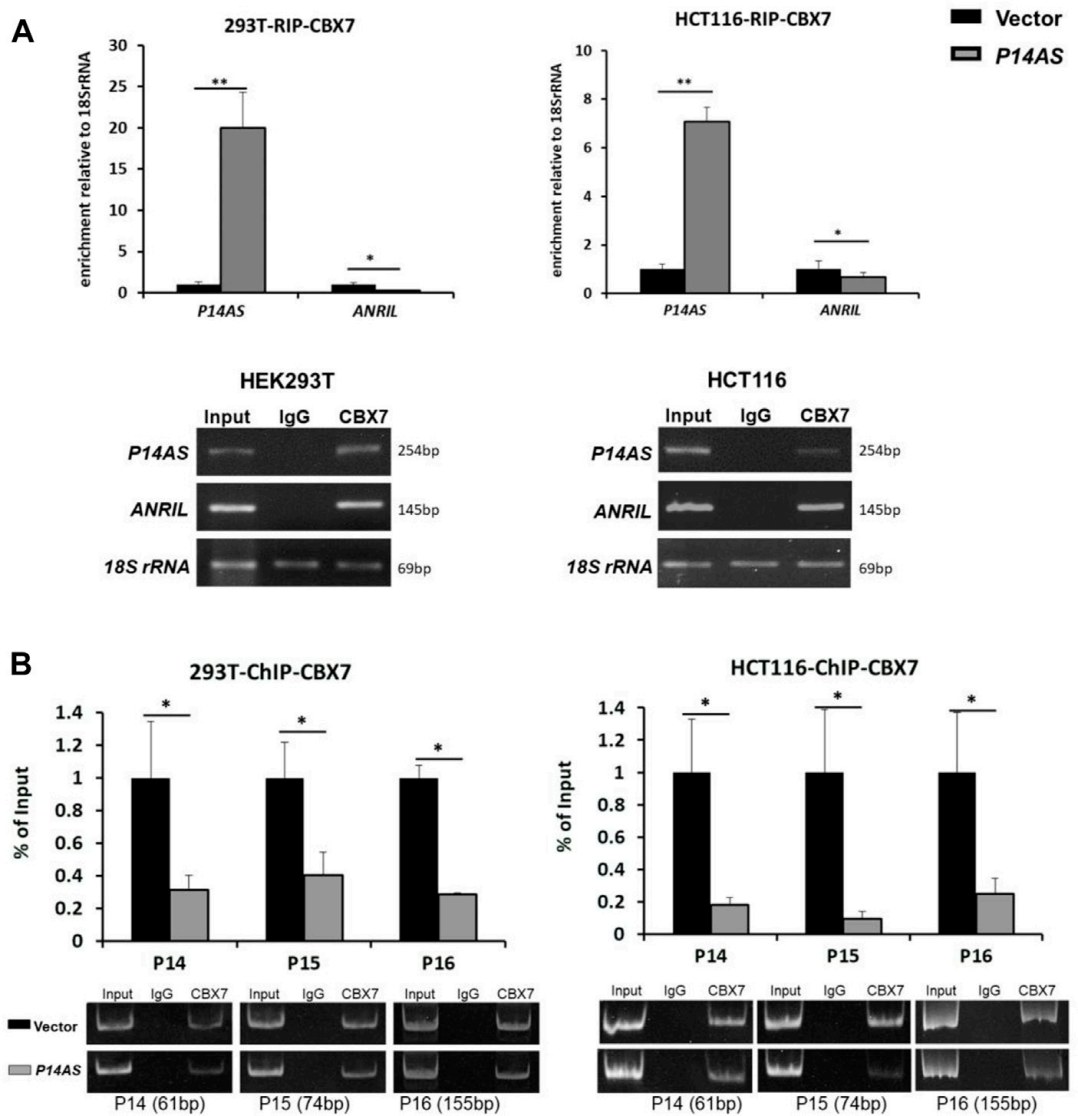
*P14AS* competitively binds to CBX7 and prevents the binding of CBX7 to *ANRIL* and to *P14* ^*ARF*^, *P15* ^*INK4B*^, *P16* ^*INK4A*^ promoters. **(A)** Levels of *P14AS* and *ANRIL* binding to endogenous CBX7, as determined by RIP-qPCR analyses; and **(B)** binding of gene promoters to endogenous CBX7, as determined by ChIP-qPCR analyses*.* The experiments were repeated using both HEK293T and HCT116 cell lines.

In the published article, there was also another error: “and” was lost in the first sentence in the discussion section. A correction has been made to **Discussion**, **Paragraph 1**.

This sentence previously stated: “It has been reported that both lncRNA *ANRIL* promoters of *P14* ^*ARF*^, *P15* ^*INK4B*^, *P16* ^*INK4A*^ can directly bind to the PcG proteins including CBX7”

The corrected sentence is as follows:

“It has been reported that both lncRNA *ANRIL* and promoters of *P14* ^*ARF*^, *P15* ^*INK4B*^, *P16* ^*INK4A*^ can directly bind to the PcG proteins including CBX7”

The authors apologize for this error and state that this does not change the scientific conclusions of the article in any way. The original article has been updated.

