# Supplementary material for: P14AS upregulates gene expression in the CDKN2A/2B locus through competitive binding to PcG protein CBX7
Source: Front Cell Dev Biol. 2022 Sep 13;10:993525. doi: 10.3389/fcell.2022.993525 (PMC9513069; doi:10.3389/fcell.2022.993525)
Supplement: Supplementary file 1 [file DataSheet1.pdf]

Supplemental Table 1. Primers and oligos used in the study

experiments	name	sequence（5‘-3’）	size	annealing temperature
**qRT-PCR & RIP-qPCR**	P14AS-F	aacggatcacatcgctcctg	254bp	58°C
	P14AS-R	tccccattcgggttacaacg		
	ANRIL-F	cagcagaaggtgggcagcagat	145bp	64°C
	ANRIL-R	ttcctcgacagggcaggcaggt		
	P14-F	gccaggggcgcccgccgctg	236bp	62°C
	P14-R	ggcccggtgcagcaccacca		
	P15-F	agtcaaccgtttcgggaggcg	168bp	62°C
	P15-R	accaccagcgtgtccaggaag		
	P16-F	gctgcccaacgcaccgaata	180bp	60°C
	P16-R	accaccagcgtgtccaggaa		
	CBX7-F	cgtcatggcctacgagga	71bp	54°C
	CBX7-R	tgggtttcggacctctctt		
	GAPDH-F	gagatggtgatgggatttc	224bp	62°C
	GAPDH-R	gaaggtgaaggtcggagt		
	ALU-F	gaggctgaggcaggagaatcg	87bp	60°C
	ALU-R	gtcgcccaggctggagtg		
	18S rRNA-F	gcttaatttgactcaacacggga	69bp	58°C
	18S rRNA-R	agctatcaatctgtcaatcctgtc		
**ChIP-qPCR**	P14-ChIP-F	gtgggtcccagtctgcagtta	61bp	56°C
	P14-ChIP-R	cctttggcaccagaggtgag		
	P15-ChIP-F	ggaacctagatcgccgatgtag	74bp	56°C
	P15-ChIP-R	tgttttacgcgtggaatgcac		
	P16-ChIP-F	cggctgggagcagggaggc	155bp	62°C
	P16-ChIP-R	gaatgtggcacccctgaagtcgc		

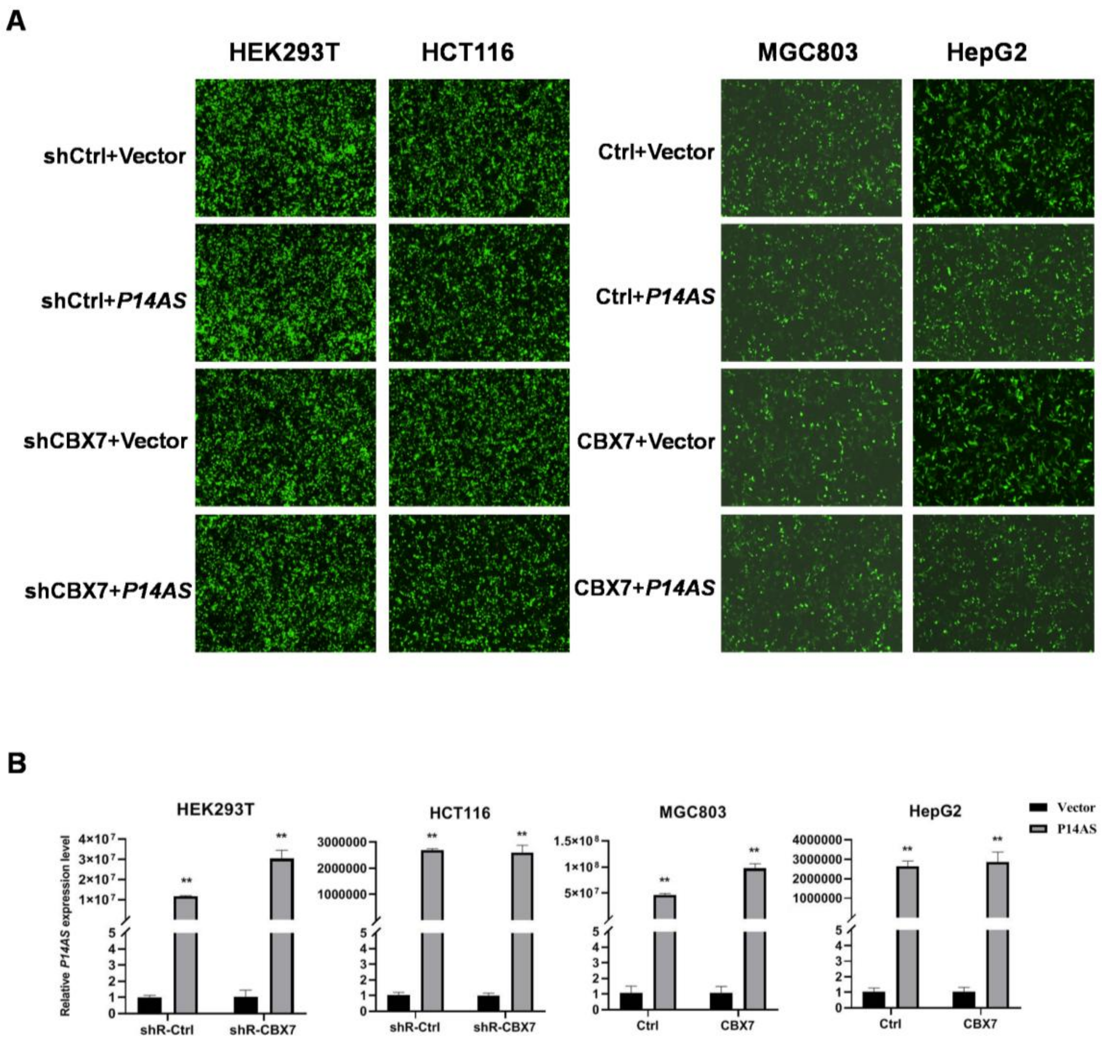

Supplemental Figure 1. The transfection efficiency of *P14AS*, *CBX7*, and shR-CBX7 expression vectors. (**A**) The green fluorescent was determined to show the transfection efficiency; (**B**) The results for *P14AS* level in the qRT-PCR analysis
